# Drinking motives among patients with alcohol use disorder: a longitudinal study

**DOI:** 10.1186/s13722-026-00656-4

**Published:** 2026-02-26

**Authors:** Lars Sjödin, Olof Molander, Stina Ingesson-Hammarberg, Anders Hammarberg

**Affiliations:** 1 Department of Social Work, Uppsala University, Uppsala, Sweden; 2 Centre for Psychiatry Research, Department of Clinical Neuroscience, Karolinska Institutet, Stockholm, Sweden; 3 Stockholm Health Care Services, Region Stockholm, Stockholm, Sweden

**Keywords:** Drinking motives, Motivation, Alcohol use disorder, AUD, Longitudinal, Clinical, Patients, Drinking, Alcohol, DMQ

## Abstract

**Background:**

This current two-year clinical study among adult patients with alcohol use disorder (AUD) examined drinking motives and their cross-sectional and longitudinal associations with alcohol consumption, severity of AUD, and other alcohol-related problems. Enhancement, coping, conformity, and social motives have been found to impact alcohol use, yet longitudinal studies on drinking motives in clinical populations are sparse.

**Methods:**

This observational study used data from a randomized controlled trial (RCT) conducted in Stockholm, Sweden, from 2017 to 2022, including 250 participants with AUD. Data from both treatment groups were included. Assessments of study outcomes were conducted at five time points: at baseline, and after 12, 26, 52, and 104 weeks. Self-report questionnaires and diagnostic interviews were used to assess drinking motives, alcohol consumption, alcohol-related problems, and mental health. Instruments used for self-reports included: The Drinking Motives Questionnaire-Revised Short Form (DMQ-R SF) and the Alcohol Use Disorders Identification Test (AUDIT). The DMQ-R SF assessed enhancement, coping, conformity, and social motives. The AUDIT assessed self-perceived alcohol problems. Interviews were conducted to assess alcohol consumption using the Timeline Follow-Back method (TLFB) and AUD using the Diagnostic and Statistical Manual of Mental Disorders (DSM-5). Generalized linear models were used to examine associations between drinking motives (DMQ R-SF) and alcohol-related outcomes (TLFB/AUDIT/AUD by the DSM-5).

**Results:**

Reductions in both drinking motives (enhancement 15.6%, coping 23.6%, conformity 15.4%, and social 17.9%) and alcohol-related outcomes (drinks per week 40.5%, number of DSM-5 criteria 50.9%, and AUDIT total score 36.3%) were observed during the study period. Across outcomes, the most prevalent drinking motives was enhancement, followed by social, coping, and conformity motives. Elevated enhancement, coping and social motives were cross-sectionally associated with worse alcohol-related outcomes at different time points, whereas higher conformity motives had links to less alcohol consumption. Higher enhancement motives at baseline predicted a larger reduction in the severity of alcohol problems from baseline to the 104-week follow-up.

**Conclusions:**

Enhancement motives were most prevalent among people with AUD, and higher enhancement motives were associated with reductions in AUD severity over time. Motive domains likely play a role in understanding changes in the severity of AUD over time.

**Trial registration:**

The original RCT study was registered retrospectively at isrctn.com (14539251), registration date 04/09/2018.

**Supplementary Information:**

The online version contains supplementary material available at 10.1186/s13722-026-00656-4.

## Introduction

Alcohol use is a large public health problem and ranks as one of the worldwide leading risk factors for ill health and mortality [1]. One way to understand why people drink alcohol despite its related risks is by focusing on the individual motivation to consume alcohol. Drinking motives are important driving factors behind alcohol use and have been suggested as one of the most proximal determinants of drinking [2, 3]. Despite this, there is a paucity of studies on drinking motives in clinical populations, and few longitudinal studies exist on this topic [4].

Theoretical models of drinking motives aim to explain why people drink and how motives are linked to specific drinking patterns [2, 3]. In such models, drinking functions as a means to achieve a desired outcome. It is further assumed that drinking motives play out as the last step in a causal chain before the drinking outcome [2]. This mediating role means that drinking motives are thought to reflect a wide range of factors influencing alcohol use. Longitudinal findings support the mediating role of drinking motives. For example, negative emotionality has been shown to predict greater drinking intensity, with coping motives acting as a mediator [5].

Most studies on drinking motives have been conducted in student samples from North America [4], conceptualizing drinking motives in a four-factor model [3, 6]. This model suggests that drinking motives reflect individuals’ internal and external motivations in regulating positive and negative emotions. Four different motives are categorized by crossing the source of the motivation (internal/external) and the emotional drive (positive/negative): enhancement (internal positive), social (external positive), coping (internal negative), and conformity (external negative). Enhancement motives reflect intentions to elevate one’s internal mood and emotional state. Social motives refer to the desire to interact and engage with others. Coping motives are focused on alleviating unpleasant feelings and emotions. Lastly, conformity motives revolve around the fear of being rejected or excluded by others [7, 8].

Previous findings in both population-based samples including adolescents, young adults, middle-aged-, older age groups, and clinical populations show that social or enhancement drinking motives are the most frequent, followed by coping motives, with conformity motives as the least frequent [9–14]. The following positive associations are typically found in younger non-clinical populations including all four motives: social motives with frequent moderate drinking, enhancement motives with heavy drinking, coping motives with alcohol-related problems, while conformity motives have been negatively linked with drinking outcomes [6–8, 15, 16]. This means that when comparing the relative strength of these four drinking motives (controlling for their mutual influence), conformity motives are associated with a decreased probability of drinking.

Although less studied, clinical populations tend to rate all four drinking motives higher than population-based samples when calculating mean scores [17–20]. In particular, coping and enhancement motives have been demonstrated to be higher among individuals with harmful alcohol use or dependence [17, 18, 20]. However, associations differ somewhat between studies. A study on adult psychiatric patients where about 4 of 10 had hazardous alcohol use showed that coping, enhancement, and social motives were all positively related to different alcohol outcomes [10], with higher quantity, frequency, binge drinking, and higher sum scores on the Alcohol Use Disorder Identification Test (AUDIT) [10, 21]. In another study on adults seeking treatment for problematic alcohol use, only coping motives were indicative of alcohol dependence [17].

These findings suggest that the links between drinking motives and alcohol outcomes in clinical populations may vary, but that coping and enhancement motives, are the most important drinking motives in individuals with AUD. Given the differences between clinical and non-clinical populations, it may be hypothesized that individual motives to drink would change as an effect of treatment for AUD. More specifically, it is plausible to assume that internal motivation (coping/enhancement) decreases with reduced alcohol problems and that the scores on the four motives during treatment for AUD gradually become more similar to those of non-clinical populations. However, this needs to be studied.

Further, most previous research on drinking motives consists of cross-sectional studies focused on adolescents or college students. Relatively few studies have been conducted on drinking motives in treatment-seeking individuals with AUD, and longitudinal designs are scarce [4]. To our knowledge, no previous study has examined individual drinking motives and their potential changes as an effect of treatment including a long-term follow-up. No study has examined how drinking motives are associated with the severity of AUD by comparing the AUDIT; a screening instrument for the identification of individuals with hazardous alcohol use and AUDs, and the diagnostic instrument Diagnostic and Statistical Manual of Mental Disorders (DSM-5), in addition to consumption measures [22]. Previous studies have shown a discrepancy between individual levels of alcohol consumption and the severity of alcohol dependence in clinical settings [23, 24], which calls for studies considering both. Understanding how drinking motives during treatment for AUD are linked to treatment outcomes is warranted. This knowledge can inform treatment providers if patients’ motives are relevant to include in assessment and treatment in AUD, with the potential to enhance the effectiveness of interventions and improve long-term outcomes.

This study aimed to explore drinking motives and their associations with long-term outcomes over two years among patients with AUD undergoing treatment. The study addressed the following research questions:

1. Do drinking motives change over time in treatment-seeking patients with AUD? Which motives are most prevalent, and do they vary across different time points?
2. How are drinking motives cross-sectionally associated with the alcohol-related outcomes of alcohol consumption, AUDIT scores, and DSM-5 AUD symptom severity over two years?
3. To what extent do baseline drinking motives predict longitudinal changes in alcohol-related outcomes (alcohol consumption, AUDIT scores, and DSM-5 AUD symptom severity) at the two-year follow-up?

## Methods

### Design

The current observational study used data collected within a randomized controlled trial (RCT) conducted at the Stockholm Centre for Dependency Disorders in Stockholm, Sweden from August 14, 2017, to December 1, 2022. The RCT aimed to investigate whether Behavioral Self-Control Training (BSCT) was superior to Motivational Enhancement Therapy (MET) in reducing weekly alcohol consumption in a sample of 250 individuals with AUD and a goal of controlled drinking [25]. The trial used a parallel-group design with a 1:1 allocation ratio, including five time points: baseline (start of treatment), and follow-ups at 12- (end of treatment), 26-, 52-, and 104 weeks post-enrollment. There were no differences between groups at the primary endpoint (26 weeks) for the reduction of mean weekly alcohol consumption. Detailed descriptions of design and outcomes from the RCT have been published elsewhere [25, 26].

### Participants and procedure

Participants were recruited among self-referred newly admitted patients at the study clinics and social media advertisements. They were subsequently assessed by a trained research coordinator using a standardized screening protocol, for the following inclusion criteria: age 18–70 years, stable housing in the Stockholm region, a diagnosis of AUD, alcohol consumption on at least 30 of the past 90 days, a stated goal of controlled drinking, i.e. a wish to reduce alcohol consumption, as opposed to a goal of abstinence (zero consumption), and willingness to provide informed consent.

Exclusion criteria were: a diagnosis of any substance use disorder other than AUD or nicotine use disorder; frequent use (more than once per week) of any illicit drug in the six months before inclusion; indicators of significant health risk associated with alcohol use, such as elevated liver enzymes (aspartate aminotransferase (AST), alanine aminotransferase (ALT), and gamma-glutamyl transferase (GGT); ongoing treatment for AUD; a major psychiatric condition, such as severe major depression or untreated bipolar disorder.

Participants meeting inclusion criteria provided informed consent to participate and were enrolled at or in conjunction with the screening session. Thereafter, they were randomly assigned to either BSCT or MET. Due to the COVID-19 pandemic, the study protocol was modified in March 2020, to maintain recruitment in the ongoing trial. Therefore, some participants (*n* = 78) had to complete baseline screening, follow-up assessments, and their treatment sessions via video using a tablet or smartphone. Self-reported data were collected by mail, with participants returning the forms in prepaid envelopes. Sensitivity analyses demonstrated no differences in the primary outcome between those who attended face-to-face treatment vs. video at 26 weeks [25, 26].

### Treatments

#### Behavioral self-control training

The Swedish adapted manual of BSCT included in the trial is a five-session cognitive and behavioral treatment focusing on skills training for achieving controlled drinking, such as goal setting, registering alcohol consumption, moderation strategies, and increasing the number of abstinent days [27, 28].

#### Motivational enhancement therapy

The Swedish MET manual consists of four sessions: an initial session providing feedback on baseline assessments, followed by three sessions of Motivational Interviewing (MI). Two optional worksheets are included to support change plan formulation and a maintenance plan [29, 30]. Both interventions were completed within a 12-week treatment period.

### Measures

#### Baseline characteristics

At baseline, participants reported sociodemographic variables such as age, gender, and occupational and marital status, as well as mental health variables including anxiety and depression symptoms. Anxiety symptoms were measured with the Generalized Anxiety Disorder Assessment (GAD-7) [31], and depression symptoms were measured with the Montgomery Asberg Depression Rating Scale-Self Reported (MADRS-S) [32].

#### Drinking motives

The Drinking Motives Questionnaire-Revised Short Form (DMQ-R SF) [6] consists of 12 items, with three items for each of the four drinking motives: enhancement (e.g. *How often do you drink because you like the feeling?*), coping *(e.g. How often do you drink to cheer up when you’re in a bad mood?)*, conformity *(How often do you drink to be liked?)* and social (e.g. *How often do you drink because it makes social gatherings more fun?*). Items are scored between 1–6, resulting in a sum score for each motive ranging between 3–18. The response options ranged from “Never” to ”Almost every drinking occasion”. Drinking motives were measured at every time point. DMQ-R SF has been tested on clinical populations in Sweden and proven to have a good model fit [10].

### Alcohol-related outcomes

#### Diagnostic interview of alcohol use disorder 

Participants underwent psychiatric diagnostics at baseline, assessed using the MINI interview based on the DSM-5, including a diagnosis of AUD [22]. DSM-5 AUD assessments were also conducted at 52 and 104 weeks, but not at the 12- or 26-week follow-ups. The interview lasted approximately one hour.

#### Assessment interview of alcohol consumption

Drinks per week were assessed at baseline and consecutive follow-up measurement points, in line with the Timeline Follow-Back method (TLFB) [33] covering the 30 days before the day of the assessment. Alcohol consumption was measured in standard drinks per day according to Swedish norms (12 g of pure ethanol). The interview took roughly one hour.

#### Self-reported alcohol problems

The AUDIT was used to measure self-perceived alcohol problems at baseline and consecutive follow-up measurement points [21]. The AUDIT was originally developed for the identification of hazardous use and dependence, and includes cut-off scores of 18 for women and 20 for men that are indicative of alcohol dependence [34].

The AUDIT comprises ten items covering three domains: consumption (items 1–3, including frequency and quantity response alternatives), dependence (items 4–6), and alcohol-related harm (items 7–10) [20]. Item 1–8 scores either 0, 1, 2, 3, or 4, while items 9–10 score either 0, 2, or 4. The total AUDIT score is calculated as the sum of all items, with a maximum score of 40.

### Statistical analyses

A statistical plan was published a priori (see AsPredicted: https://aspredicted.org/4nf2c.pdf). To adopt a longitudinal approach, we included all measurement points including baseline drinking motives. We also included two additional outcome variables—the AUDIT and DSM-5 criteria—to incorporate clinically relevant measures beyond alcohol consumption. The original statistical plan incorporated a stepwise regression approach controlling for age, gender, depression, and anxiety as covariates. Along the review process, analyses were revised for parsimony to use only multivariate regressions to examine associations between drinking motives and alcohol-related outcomes, excluding these covariates. This streamlined approach was applied to improve clarity and reduce model complexity, resulting in more focused models that prioritized drinking motives and alcohol-related variables. The overall pattern of the results remained robust regardless of analysis procedure.

Across measures, missing data were the following at baseline (≈ 1%), 12 weeks (≈ 15%), 26 weeks (≈ 20%), 52 weeks (≈ 30%), and 104 weeks (≈ 40%). These missing data were handled using multiple imputations [35] with predictive mean matching (200 datasets, 30 iterations), and analyses were performed across all imputed datasets following Rubin’s rules [36]. Supplementary Table 1 presents demographic variables for the original and imputed datasets.

The following thresholds were utilized for the outcomes of this study: For alcohol consumption, the mean weekly number of drinks was calculated by dividing the total number of standard drinks consumed in the past 30 days by the number of weeks (4.29), as assessed by the TLFB. Low versus high consumption was determined using dichotomized drinks-per-week variables, based on median splits of the weekly number of drinks at baseline at each subsequent measurement point. For the AUDIT cut-off, a total score of 18 for women and 20 for men was used, corresponding to alcohol dependence [34]. For DSM-5 criteria, the number of criteria fulfilled was used, along with symptom severity categorized as mild (2–3 criteria), moderate (4–5 criteria), or severe AUD (6–11 criteria) [22].

Changes in alcohol-related outcomes were evaluated as the difference between baseline and the final follow-up at 104 weeks. Change in alcohol consumption was operationalized as a dichotomous variable (low vs. high change) based on the median percentage reduction (median = 48.5%), with baseline to 104 weeks percental change coded as a positive value. Likewise, change in AUDIT score was dichotomized according to the median percentage reduction (median = 35.0%), with percental change coded as a positive value. Change in DSM-5 criteria was quantified as the absolute reduction in the number of criteria met, reflecting a positive value.

Descriptive statistics were reported as frequencies, percentages, means, and standard deviations. Primary motives were identified by determining the DMQ motive with the highest score at each measurement point, presented in both frequencies and percentages, with individual trajectories visualized using a Sankey diagram. Mean differences in drinking motives across alcohol-related outcomes were examined using t-tests at each measurement point. P-values < 0.05 were considered significant.

Cross-sectional associations between drinking motives and alcohol-related outcomes were analyzed using multivariate regression models. Finally, predictors of longitudinal changes in alcohol-related outcomes – drinking motives - were assessed using generalized linear regression. All analyses were conducted using R Studio [37] and utilized key packages, including mice, dplyr, and psych.

## Results

Baseline demographics and clinical characteristics of the sample are detailed in Table 1. Briefly, most of the patients had a moderate or severe AUD, reported alcohol consumption of approximately 23 standard drinks per week, were on average slightly above 50 years of age, and about half of them were female. Self-reported anxiety and depressive symptoms were low in the sample, indicating sub-diagnostic or mild symptom severity [31, 32].

### Drinking motives over time

**Fig. 1:**
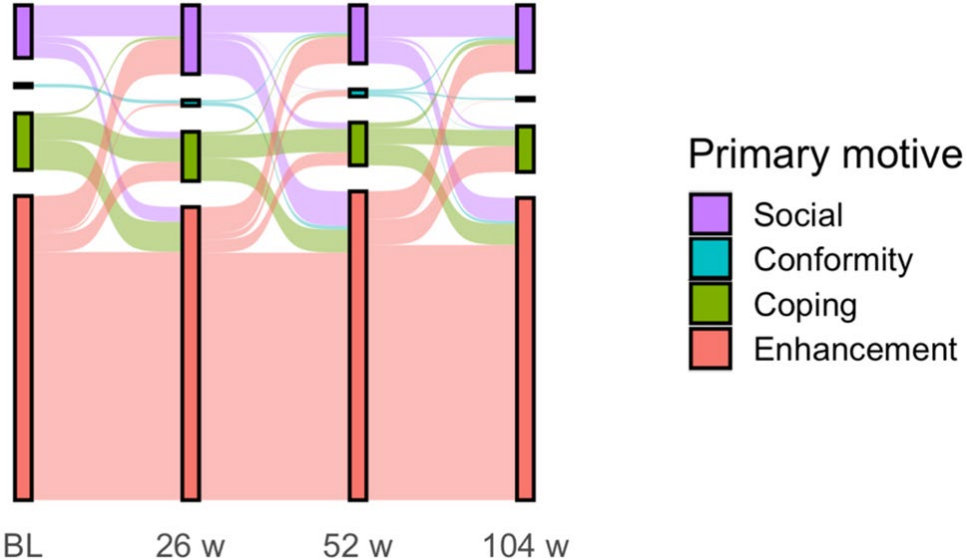
Sankey Plot of primary drinking motives of people with AUD seeking controlled drinking at study entry, 26, 52, and 104 weeks in Stockholm, Sweden: 2017–2022. Missing data were handled using multiple imputation; analyses were based on a total sample size of 250. Details on missing data are provided in Supplementary Table 1

At baseline, the drinking motives with the highest reported mean score was enhancement, followed by social, coping, and conformity (see Table 1). Throughout the two-year follow-up period, this ranking remained consistent, though all drinking motives scores continuously decreased over time. Reductions from baseline to the 104-week follow-up were highest for coping and lowest for conformity motives. Enhancement was the most frequently reported primary motives throughout the study, with similar proportions observed at follow-ups. A sensitivity analysis revealed no significant mean differences in drinking motives based on treatment condition at any of the follow up time-points. Individual participants generally remained consistent in their primary motives over the two years, with relatively few shifting their primary motives across different measurement points (see Fig. 1).

All alcohol- outcomes decreased from baseline to the 104-week follow-up. Weekly alcohol consumption, the number of fulfilled DSM-5 criteria, and the proportion of participants exceeding the AUDIT cut-off for alcohol dependence, decreased between a quarter and eight-tenths throughout the study (see Table 1).

**Table 1:** Characteristics and drinking motives of people with AUD seeking controlled drinking in Stockholm, Sweden: 2017–2022 (*N* = 250)

	Measurement point					Change baseline to 104 weeks
	Baseline	12 weeks	26 Weeks	52 weeks	104 weeks	
Demographic characteristics	n (%)					M (%)
Male	131 (52.4%)	-	-	-	-	-
Female	119 (47.6%)	-	-	-	-	-
Age, M (Sd)	51.8 (11.0)	-	-	-	-	-
Drinking motives	Range 3–18 - M (Sd)					
Enhancement	12.2 (3.2)	11.1 (3.4)	10.7 (3.5)	10.3 (3.7)	10.3 (3.6)	-1.9 (15.6%)
Coping	8.9 (3.6)	7.4 (3.5)	7.2 (3.1)	6.7 (3.0)	6.8 (3.1)	-2.1 (23.6%)
Conformity	5.2 (2.5)	4.9 (2.3)	4.9 (2.3)	4.6 (2.3)	4.4 (2.0)	-0.8 (15.4%)
Social	9.5 (3.9)	8.7 (3.6)	8.4 (3.6)	8.0 (3.7)	7.8 (3.6)	-1.7 (17.9%)
Primary drinking motives	n (%)					
Enhancement	182 (72.9%)	184 (73.8%)	176 (70.3%)	185 (74.0%)	181 (72.5%)	-
Coping	34 (13.6%)	25 (9.9%)	30 (11.8%)	26 (10.3%)	27 (10.9%)	-
Conformity	2 (0.8%)	5 (2.1%)	3 (1.4%)	4 (1.7%)	1.5 (0.6%)	-
Social	32 (12.6%)	36 (14.2%)	41 (16.5%)	35 (14.0%)	40 (16.0%)	-
Alcohol-related characteristics	M (Sd)					
Drinks per week	22.7 (13.1)	14.4 (9.5)	13.2 (8.8)	12.7 (9.6)	13.5 (10.5)	-9.2 (40.5%)
Number of DSM-5 criteria, Range 0–11 - M (Sd)	5.3 (2.0)	-	-	2.9 (2.0)	2.6 (2.2)	-2.7 (50.9%)
AUDIT total score Range 0–40 – M (sd)	19.0 (5.8)	15.8 (5.9)	14.4 (5.5)	12.4 (5.5)	12.1 (5.2)	-6.9 (36.3%)
AUDIT cutoff (> 18/20 for women/men), n (%)	136 (54.3%)	75 (30%)	56 (22.2%)	33 (13.3%)	28 (11.1%)	-108 (79.4%)
Psychiatric characteristics						
Depression, MADRS, Range 0– 54, total score	9.3 (6.9)	8.2 (6.9)	7.7 (6.7)	7.3 (6.4)	7.3 (7.0)	-2.0 (21.5%)
Anxiety, GAD-7 Range 0– 21, total score	3.4 (3.8)	2.9 (3.7)	3.1 (4.0)	2.8 (3.8)	2.8 (3.5)	-0.6 (17.6%)

Note: Missing data were handled using multiple imputation; analyses were based on a total sample size of 250. Details on missing data are provided in Supplementary Table 1

The Sankey Plot (Fig. 1) illustrates individual trajectories of primary motives (highest mean score) across measurement points. As can be seen, the bulk of enhancement drinkers remained to have enhancement as their primary motive throughout the study period. However, transitions in primary motive also occurred - for example, about half of those with coping as their primary motive at baseline switched at each time point, and the majority with a social primary motive transitioned after 26 weeks.

### Differences in drinking motives by alcohol-related outcomes

Drinking motives were explored in relation to outcomes across measurement points. The results showed that significantly higher scores on enhancement and coping motives were observed in individuals with weekly alcohol consumption above the median. This was also true for those with AUDIT scores above the cut-off indicating alcohol dependence, and for those with a higher severity level in DSM-5 AUD, compared to those with lower scores on these alcohol-related measures. Overall, this pattern was observed both at baseline and at follow-up assessments post-treatment. Higher conformity and social motives showed fewer significant mean differences in relation to alcohol consumption and AUDIT scores above the 18/20 cut-off, compared to enhancement and coping motives (see Table 2).

**Table 2:** Mean differences in drinking motives by alcohol-related outcomes among people with AUD seeking controlled drinking in Stockholm, Sweden: 2017–2022 (*N* = 250)

DMQ subscales, Range 3–18 - M (Sd)	Drinks per week (median split^1^)		AUDIT cut-off (18/20 for women/men)		DSM-5, Alcohol Use Disorder (symptom severity^2^)		
	Low	High	Below	Above	Mild	Moderate	Severe
**Enhancement**							
Baseline	11.9 (3.4)	12.5 (3.0)	11.1 (3.2)	13.2 (2.8)***	10.0 (2.98)	12.3 (3.27)***	13.2 (2.65)***
12 weeks	10.1 (3.4)	12.1 (3.0)***	10.2 (3.3)	13.0 (2.7)***	x	x	x
26 weeks	10.1 (3.7)	11.2 (3.2)*	10.2 (3.6)	12.2 (2.7)***	x	x	x
52 weeks	9.5 (3.7)	11.3 (3.4)***	10.1 (3.6)	11.9 (3.6)*	8.36 (3.15)	10.4 (3.62)***	11.3 (3.57)***
104 weeks	9.4 (3.8)	11.1 (3.3)*	10.1 (3.6)	11.4 (3.3)	8.43 (3.27)	10.5 (3.63)***	11.0 (3.47)*
**Coping**							
Baseline	8.7 (3.6)	9.1 (3.7)	7.7 (3.3)	9.9 (3.6)***	6.31 (2.54)	8.83 (3.28)***	10.2 (3.70)***
12 weeks	6.9 (3.2)	7.9 (3.7)*	6.5 (3.0)	9.4 (3.7)***	x	x	x
26 weeks	6.8 (2.8)	7.5 (3.3)	6.6 (2.8)	9.1 (3.4)***	x	x	x
52 weeks	6.0 (2.8)	7.4 (2.9)**	6.5 (2.8)	8.2 (3.2)**	5.16 (2.05)	6.36 (2.76)***	7.68 (3.10)***
104 weeks	5.9 (2.8)	7.6 (3.2)***	6.3 (2.9)	10.1 (2.9)***	4.97 (2.27)	6.54 (2.83)***	7.79 (3.22)***
**Conformity**							
Baseline	5.4 (2.7)	5.0 (2.3)	4.6 (2.3)	5.7 (2.7)**	4.30 (2.17)	5.68 (2.58)***	5.34 (2.57)***
12 weeks	4.9 (2.3)	5.0 (2.2)	4.6 (2.0)	5.7 (2.7)**	x	x	x
26 weeks	4.8 (2.2)	5.0 (2.3)	4.9 (2.3)	5.1 (2.3)	x	x	x
52 weeks	4.7 (2.4)	4.5 (2.1)	4.5 (2.2)	5.4 (2.8)	4.05 (1.56)	4.87 (2.39)	4.67 (2.43)***
104 weeks	4.5 (1.9)	4.4 (2.0)	4.4 (2.0)	4.4 (1.9)	3.88 (1.52)	4.80 (2.17)	4.43 (1.93)***
**Social**							
Baseline	9.4 (4.0)	9.6 (3.8)	8.3 (3.5)	10.5 (3.8)***	8.13 (3.81)	9.67 (3.53)	10.1 (3.94)***
12 weeks	8.2 (3.6)	9.2 (3.6)	8.0 (3.4)	10.4 (3.6)***	x	x	x
26 weeks	7.8 (3.5)	8.9 (3.6)*	8.1 (3.6)	9.2 (3.6)	x	x	x
52 weeks	7.7 (3.7)	8.3 (3.7)	7.8 (3.7)	9.2 (4.0)	7.01 (3.29)	7.99 (3.50)	8.45 (4.03)***
104 weeks	7.5 (3.5)	8.1 (3.6)	7.8 (3.5)	8.0 (3.9)	6.98 (3.64)	8.01 (3.45)***	8.06 (3.56)***

Note. The table presents differences in drinking motives by categorical alcohol-related outcomes across measurement points

* = significant *p* ≤ .05; ** = *p* ≤ .01; *** = *p* ≤ .01; x = DSM-5, Alcohol Use Disorder was not assessed at 12 and 26 weeks. For DSM-5 outcomes, mild AUD was compared with moderate severity, and moderate symptom severity with severe AUD

^1^Based on median splits of the weekly number of drinks at each measurement point (baseline Mdn = 19.4; 12-week follow-up Mdn = 12.2; 26-week follow-up Mdn = 11.0; and 52-week follow-up Mdn = 10.5)

^2^According to the DSM-5 diagnostic criteria; 2–3, 4–5 or 6–11 criteria fulfilled, mild, moderate or severe alcohol use disorder, respectively

^3^ Missing data were handled using multiple imputation; analyses were based on a total sample size of 250. Details on missing data are provided in Supplementary Table 1

### Cross-sectional associations between drinking motives and alcohol-related outcomes

Associations between drinking motives and alcohol-related outcomes were examined at each measurement point using multivariable models (see Table 3). Overall, higher enhancement and coping motives were associated with greater weekly alcohol consumption, higher proportions exceeding AUDIT dependence cut-offs, and more DSM-5 AUD symptoms, whereas conformity motives showed negative associations with greater weekly alcohol consumption after 26 weeks and onwards. Social motives were linked to a higher weekly consumption only at one time point.

**Table 3:** Cross-sectional significant multivariate associations between drinking motives and alcohol-related outcomes among people with AUD seeking controlled drinking in Stockholm, Sweden: 2017–2022 (*N* = 250)

Odds Ratio (95 CI%)	Drinks per week (low, high^1^)	AUDIT cut-off (18/20 for women/men)	DSM-5, Alcohol Use Disorder (number of criteria)
Drinking motive	Baseline predictors and outcomes		
Enhancement	-	1.03 (1.01–1.06) *p*≤.01	1.21 (1.11–1.31) *p*≤.001
Coping	-	1.03 (1.01–1.05) *p*≤.001	1.19 (1.12–1.27) *p*≤.001
Conformity	-	-	-
Social	-	-	-
	**12 weeks predictors and outcomes**		
Enhancement	1.03 (1.00-1.05) *p*≤.05	-	x
Coping	-	1.03 (1.01–1.05) *p*≤.001	x
Conformity	-	-	x
Social	-	-	x
	**26 weeks predictors and outcomes**		
Enhancement	-	-	x
Coping	-	1.03 (1.02–1.05) *p*≤.001	x
Conformity	0.96 (0.93–0.99) *p*≤.05	-	x
Social	1.02 (1.00-1.05) *p*≤.05	-	x
	**52 weeks predictors and outcomes**		
Enhancement	-	-	-
Coping	-	1.02 (1.00 -1.03) *p*≤.05	-
Conformity	0.97 (0.94-1.00) *p*≤.05	-	-
Social	-	-	-
	﻿**104 weeks predictors and outcomes**		
Enhancement	-	-	-
Coping	-	1.03 (1.01–1.04) *p*≤.01	1.15 (1.04–1.27) *p*≤.01
Conformity	0.97 (0.94-1.00) *p*≤.05	-	-
Social	-	-	-

Note. The table present significant associations between drinking motives and alcohol-related outcomes across measurement points. The multivariate analyses included all four drinking motives

A dash (-) indicates a non-significant result

x = DSM-5, Alcohol Use Disorder was not assessed at 12 and 26 weeks

^1^Based on median splits of the weekly number of drinks at each measurement point (baseline Mdn = 19.4; 12-week follow-up Mdn = 12.2; 26-week follow-up Mdn = 11.0; and 52-week follow-up Mdn = 10.5)

^1^ Missing data were handled using multiple imputation; analyses were based on a total sample size of 250. Details on missing data are provided in Supplementary Table 1

### Initial drinking motives and changes in alcohol-related outcomes

Lastly, we examined the predictive value of baseline drinking motives on changes in alcohol-related outcomes from baseline to the 104-week follow-up (see Table 4) using multivariate regression analyses. Enhancement motives were the only significant predictors of longitudinal change, with higher baseline enhancement scores associated with greater reductions in AUDIT scores and DSM-5 AUD symptom severity over two years.

**Table 4:** Baseline drinking motives predicting longitudinal changes in alcohol-related outcomes over 104 weeks among individuals with AUD seeking controlled drinking in Stockholm, Sweden: 2017–2022 (*N* = 250)

Baseline Predictors	Drinks per week (percental change, low, high)	AUDIT (change in total score)	DSM-5, Alcohol Use Disorder (change in the number of criteria)
Enhancement	-	1.38 (1.02–1.87) *p* ≤ .05	1.16 (1.02–1.31) *p* ≤ .05
Coping	-	-	-
Conformity	-	-	-
Social	-	-	-

Note. The table presents multivariable regression analyses of baseline predictors for changes in alcohol-related outcomes. A dash (-) indicates a non-significant result

^1^Changes in AUDIT and DSM-5 baseline to 104 weeks outcomes were coded as positive values; therefore, effect estimates greater than 1 reflect a greater reduction

^2^Missing data were handled using multiple imputation; analyses were based on a total sample size of 250. Details on missing data are provided in Supplementary Table 1

## Discussion

In this study, drinking motives in patients with AUD were investigated across two years, before and after receiving 12 weeks of treatment aimed at reducing alcohol consumption. Additionally, we examined cross-sectional and longitudinal associations between drinking motives and alcohol-related outcomes.

In summary, we found that the most prevalent motives were enhancement, followed by social, coping, and conformity. This rank order remained consistent across all data points. Further, all four drinking motives decreased throughout the study period, as did all three alcohol-related treatment outcomes. A main finding was that enhancement and coping motives, were cross-sectionally positively associated with both alcohol consumption and alcohol problems. This means that higher enhancement and coping motives scores were related to higher alcohol consumption and more severe alcohol-related problems. However, these associations varied in terms of which outcome was significant at each time point. Conformity motives showed an opposite link by being negatively associated with alcohol consumption. Higher scores on enhancement motives at baseline predicted larger reductions in AUDIT scores and DSM-5 AUD symptom severity after two years.

Although all four drinking motives were present among participants, enhancement motives were most dominant, as shown by both the mean scores and the proportion of participants who identified enhancement as their primary motive. This suggests that individuals with AUD mainly drink for the pleasurable effects of alcohol and to enjoy the sensation of drunkenness, driven by positive emotions. This partly contrasts with findings in general adult populations, where social drinking motives are typically the most common or on pair with enhancement motives [14, 38]. However, the observation of enhancement motives being most prevalent in our sample shows similarities with other clinical studies where enhancement has also been found to be the most common drinking motive, followed by social or coping motives [10, 17]. This finding may challenge the self-medication model, suggesting that drinking to cope plays a key role in alcohol problems. More recent findings have highlighted that reward, and *liking* drives the development and maintenance of AUDs [39].

Studies of general population samples report that drinking motives differ somewhat between countries [40–42]. Reports on drinking motives among the general Swedish adult population show that enhancement motives for drinking are the most common, followed by social motives, while other motives are less prevalent [43]. Enhancement motives have also shown to be highly prevalent in the Swedish general youth population and have the strongest link to heavier consumption [9]. Considering the *dry drinking culture* in Nordic countries [44] characterized by infrequent high-volume drinking, the Swedish population may be more prone to drinking for enhancement motives, hence reflected in our results for this clinical sample.

Second, all drinking motives declined across the two years. However, we also observed that the mean rank order in different drinking motives prevailed to a large degree during the study period. The consistency in rank order suggests that motives might be somewhat robust, rather than highly flexible states susceptible to change as suggested by Crutzen and colleagues in a study of adult heavy drinkers [45]. Despite many transitions, the ranking of primary motives also remained highly stable at the individual level. However, we did find that mean scores for coping motives were reduced the most compared to the other motives. Overall, patients in the study reduced their alcohol consumption from hazardous levels, with many sustaining low-risk drinking over time [25]. We also observed a reduction in anxiety and depression symptoms. Since lower alcohol consumption is expected to decrease depressive symptoms, coping motives could account for this relationship, thereby reducing the desire to drink as a coping mechanism.

Third, we found that higher enhancement and coping motives were cross-sectionally associated with higher alcohol consumption, higher AUDIT scores, and greater DSM-5 AUD symptom severity at different time points over the study period. Other studies on clinical samples have shown similar results, although coping motives appeared to have been most important in these studies [10, 17]. Previous findings show that both coping motives and enhancement motives tend to be higher among people with harmful alcohol use and dependence than among those with lower consumption [14, 17–20]. A meta-analysis of cross-sectional and longitudinal studies on drinking motives and drinking outcomes found that enhancement and coping motives had the strongest associations with more drinking problems [4]. Given the addictive nature of alcohol [46], one theoretical interpretation is that alcohol use strengthens these internal motives, consequently making them more prevalent among individuals with alcohol problems. We found that the strong internal desire to drink in people with AUD decreased when they reduced their consumption. Social and conformity motives were associated with alcohol consumption after reductions in drinking. This suggests that external motivation gained importance after treatment. When individuals drank less, social motives were linked to higher weekly drinking, while conformity motives were protective with links to lower weekly drinking.

Lastly, longitudinal findings showed that enhancement motives at baseline predicted change in DSM-5 AUD severity and AUDIT scores from baseline to the two-year follow-up when controlling for overlapping motives. This indicates that the level of enhancement motives at the beginning of treatment influenced the extent of reductions in alcohol problems over time. In contrast, a recent 12-week study on treatment-seeking individuals with an AUD found that those drinking for relief (i.e. coping/conformity motives) had higher alcohol use (TFLB), greater heavy drinking, more alcohol craving, and fewer abstinent days compared to those drinking for reward (i.e. social/enhancement motives) [47]. While drinking motives have shown great potential in understanding drinking patterns in observational studies, their usefulness in treatment for alcohol problems remains understudied.

### Strengths and limitations

The major strength of this study is the large clinical sample followed at five time-points over two years. Few studies have examined drinking motives longitudinally and most studies within the field have used samples in the general population. We used diagnostic interviews for the assessment of AUD and gold-standard instruments, such as the timeline follow-back for the measurement of alcohol consumption, to ensure reliable and accurate assessments.

Given the heterogeneity of AUD, it can be expected that drinking motives may differ between individuals with different levels of severity of the disorder. The current trial did not allow patients to have severe mental health issues and frequent substance use. Rather, the sample was characterized by high social functioning and a low level of psychiatric comorbidities. This may have resulted in partly homogenous results of drinking motives, and lower endorsements of coping motives compared to more severely affected samples with AUD. There was substantial data loss, particularly at the two-year follow-up, which represents a limitation of our study. Given the study design, which included a two-year follow-up period, some degree of participant attrition was expected. However, the loss of participants may have limited the availability of important information regarding potential changes in drinking motives over time. To address this limitation and reduce potential selection bias, we applied appropriate statistical methods, specifically multiple imputation.

Lastly, we cannot rule out that drinking motives differ between individuals with AUD who aim for abstinence and those pursuing controlled drinking. It would have been of clinical interest to examine how motives change over time in patients who strive for abstinence. Due to our study design, our inferences are limited to patients with a controlled drinking goal.

### Clinical implications

From a patient-centered perspective, drinking motives are intuitive and close to patients’ descriptions of what is driving their problematic drinking behaviors. Our findings highlight the importance of identifying patients who consume alcohol for enhancement and coping purposes. Higher scores on these drinking motives are linked to increased severity of alcohol-related problems and higher alcohol consumption in individuals with AUD. Patients’ motives also indicate suitable treatment targets, for example, an emphasis on alternative, non-drinking activities that increase positive mood, and emotional well-being in patients with coping motives. In addition, understanding individual drinking motives can inform the use of personalized motivational strategies, such as exploring discrepancies between the expected and actual outcomes of drinking, or reinforcing intrinsic goals that conflict with alcohol use. Patients’ drinking motives can hence guide the content in treatment to identify key targets that would challenge the reinforcers of alcohol consumption.

## Conclusions

In persistent rank order, the level of drinking motives was continuously reduced across two years, along with reduced alcohol consumption and related problems. Enhancement motives were consistently the most common, followed by social, coping, and conformity motives. Systematic differences in drinking motives were found by the level of alcohol consumption and by severity of alcohol problems indicating dependence among patients with AUD. Enhancement and coping motives were repeatedly related to concurrent worse alcohol-related outcomes, although associations were somewhat inconsistent across time points and outcomes. Greater enhancement motives predicted future reductions in alcohol problems across two years.

## Supplementary Information

Below is the link to the electronic supplementary material.

Supplementary Material 1

## Data Availability

The datasets used and analyzed in the current study are not publicly available but can be provided by the last author upon reasonable request.

## Abbreviations

ALT: Alanine Transaminase
AST: Aspartate Transaminase
AUD: Alcohol Use Disorder
AUDIT-C: Alcohol Use Disorders Identification Test - Consumption
BSCT: Behavioral Self-Control Training
COVID-19: Corona Virus Disease 2019
DSM: Diagnostic and Statistical Manual of Mental Disorders
DMQ-R SF: Drinking Motives Questionnaire-Revised Short Form
GAD: Generalized Anxiety Disorder
GGT: Gamma-Glutamyl Transferas
MADRS-S: Montgomery Asberg Depression Rating Scale - Self Reported
MET: Motivational Enhancement Therapy
MI: Motivational Interviewing
MINI: Mini International Neuropsychiatric Interview
RCT: Randomized Controlled Trial
TLFB: Timeline Follow-Back
