# Supplementary material for: Drinking motives among patients with alcohol use disorder: a longitudinal study
Source: Addict Sci Clin Pract. 2026 Feb 26;21:27. doi: 10.1186/s13722-026-00656-4 (PMC12949512; doi:10.1186/s13722-026-00656-4)
Supplement: Supplementary file 1 — Supplementary Material 1 [file 13722_2026_656_MOESM1_ESM.docx]

**Supplementary Table 1**

*Participant characteristics and clinical descriptives, original and imputed datasets*

|  | Baseline | | 12 weeks | | 26 Weeks | | 52 weeks | | 104 weeks | |
| --- | --- | --- | --- | --- | --- | --- | --- | --- | --- | --- |
|  | Original  Complete data  n=249 | Imputed  n=250 | Original  Complete data  n=217 | Imputed  n=250 | Original  Complete data  n=199 | Imputed  n=250 | Original  Complete data  n=169 | Imputed  n=250 | Original  Complete data  n=151 | Imputed  n=250 |
| Demographic characteristics | n (%) | |  |  | |  | | | |  |
| Male | 131 (52.4%) | 131 (52.4%) | - | - | - | - | - | - | - | - |
| Female | 119 (47.6%) | 119 (47.6%) | - | - | - | - | - | - | - | - |
| Age, M (Sd) | 51.8 (11.0) | 51.8  (11.0) | - | - | - | - | - | - | - | - |
| Drinking motives |  | M (Sd) | | | | | | | | |
| Enhancement | 12.2 (3.2) | 12.2  (3.2) | 11.0 (3.4) | 11.1  (3.4) | 10.7 (3.5) | 10.7  (3.5) | 10.3 (3.7) | 10.3  (3.7) | 10.2 (3.6) | 10.3  (3.6) |
| Coping | 8.9  (3.7) | 8.9  (3.6) | 7.3  (3.5) | 7.4  (3.5) | 7.0  (3.0) | 7.2  (3.1) | 6.4  (2.8) | 6.7  (3.0) | 6.3  (2.9) | 6.8  (3.1) |
| Conformity | 5.2  (2.5) | 5.2  (2.5) | 5.0  (2.3) | 4.9  (2.3) | 5.0  (2.3) | 4.9  (2.3) | 4.6  (2.3) | 4.6  (2.3) | 4.5  (2.0) | 4.4  (2.0) |
| Social | 9.5  (3.9) | 9.5  (3.9) | 8.7  (3.7) | 8.7  (3.6) | 8.5  (3.6) | 8.4  (3.6) | 7.9  (3.6) | 8.0  (3.7) | 7.8  (3.5) | 7.8  (3.6) |
| Primary drinking  motives |  | n (%) | | | | | | | | |
| Enhancement | 182 (73.1%) | 182 (72.9%) | 160 (73.7%) | 184 (73.8%) | 142 (71.4%) | 176 (70.3%) | 133 (78.7%) | 185 (74.0%) | 116 (76.8%) | 181 (72.5%) |
| Coping | 34 (13.7%) | 34  (13.6%) | 20 (9.2%) | 25  (9.9%) | 20 (10.1%) | 30  (11.8%) | 12 (7.1%) | 26  (10.3%) | 10 (6.6%) | 27  (10.9%) |
| Conformity | 2  (0.8%) | 2  (0.8%) | 5  (2.3%) | 5  (2.1%) | 3  (1.5%) | 3  (1.4%) | 3  (1.8%) | 4  (1.7%) | 1  (0.7%) | 1.5  (0.6%) |
| Social | 31  (12.4%) | 32  (12.6%) | 32 (14.7%) | 36  (14.2%) | 34 (17.1%) | 41  (16.5%) | 21 (12.4%) | 35  (14.0%) | 24 (15.9%) | 40  (16.0%) |
| Alcohol-related characteristics |  | M (Sd) | | | | | | | | |
| Drinks per week | 22.7 (13.1) | 22.7  (13.1) | 14.1 (9.4) | 14.4  (9.5) | 13.2 (8.9) | 13.2  (8.8) | 12.3 (9.2) | 12.7  (9.6) | 13.1  (10) | 13.5  (10.5) |
| Number of  DSM-5 criteria,  M (Sd) | 5.3  (2.0) | 5.3  (2.0) | - | - | - | - | 2.7  (2.0) | 2.9  (2.0) | 2.5  (2.2) | 2.6  (2.2) |
| AUDIT total  score | 19.0 (5.8) | 19.0  (5.8) | 15.6 (5.9) | 15.8  (5.9) | 14.3 (5.5) | 14.4  (5.5) | 12.3 (5.4) | 12.4  (5.5) | 11.9 (5.0) | 12.1  (5.2) |
| AUDIT cutoff  (>18/20 for  women/men),  n (%) | 133 (54.1%) | 136 (54.3%) | 60 (28.3%) | 75  (30%) | 42 (21%) | 56  (22.2%) | 19 (11.1%) | 33  (13.3%) | 11 (7.7%) | 28  (11.1%) |
| Psychiatric characteristics |  | M (Sd) | | | | | | | | |
| Depression,  MADRS  total score | 9.3  (6.9) | 9.3  (6.9) | 8.1  (6.8) | 8.2  (6.9) | 7.5  (6.6) | 7.7  (6.7) | 6.5  (6.1) | 7.3  (6.4) | 6.6  (6.7) | 7.3  (7.0) |
| Anxiety, GAD-7  total score | 3.4  (3.8) | 3.4  (3.8) | 2.8  (3.6) | 2.9  (3.7) | 3.0  (3.9) | 3.1  (4.0) | 2.3  (3.5) | 2.8  (3.8) | 2.4  (3.2) | 2.8  (3.5) |
